# Improving community case management of diarrhoea and pneumonia in district Badin, Pakistan through a cluster randomised study—the NIGRAAN trial protocol

**DOI:** 10.1186/s13012-014-0186-9

**Published:** 2014-12-10

**Authors:** Fauziah Rabbani, Aftab Akbar Ali Mukhi, Shagufta Perveen, Xaher Gul, Saleem Perwaiz Iqbal, Shamim Ahmed Qazi, Iqbal Azam Syed, Khalid Hussain Shaikh, Wafa Aftab

**Affiliations:** Department of Community Health Sciences, The Aga Khan University, Stadium Road, 3500, Karachi, 74800 Pakistan; Department of Maternal, Newborn, Child and Adolescent Health, World Health Organization, 20 Avenue Appia, 1211 Geneva 27, Switzerland; Department of Health, Government of Sindh, 6th floor, New Sindh Secretariat, Karachi, Pakistan

**Keywords:** Community case management, Pneumonia and diarrhoea, LHW programme, Supervision, Implementation research, Pakistan

## Abstract

**Background:**

Diarrhoea and pneumonia contribute 30% of deaths in children under 5 in Pakistan. Pakistan’s Lady Health Workers Programme (LHW-P) covers about 60% of the population but has had little impact in reducing morbidity and mortality related to these major childhood killers. An external evaluation of the LHW-P suggests that lack of supportive supervision of LHWs by lady health supervisors (LHSs) is a key determinant of this problem. Project NIGRAAN aims to improve knowledge and skills of LHWs and community caregivers through supervisory strategies employed by LHSs. Ultimately, community case management (CCM) of childhood pneumonia and diarrhoea will improve.

**Methods/Design:**

NIGRAAN is a cluster-randomised trial in District Badin, Pakistan. There are approximately 1100 LHWs supervised by 36 LHSs in Badin. For this study, each LHS serves as a cluster. All LHSs working permanently in Badin who regularly conduct and report field visits are eligible. Thirty-four LHSs have been allocated to either intervention or control arms in a ratio of 1:1 through computer-generated simple randomisation technique. Five LHWs from each LHSs are also randomly picked. All 34 LHSs and 170 LHWs will be actively monitored. The intervention consists of training to build LHS knowledge and skills, clinical mentorship and written feedback to LHWs. Pre- and post-intervention assessments of LHSs, LHWs and community caregivers will be conducted via focus group discussions, in-depth interviews, knowledge assessment questionnaires, skill assessment scorecards and household surveys.

Primary outcome is improvement in CCM practices of childhood diarrhoea and pneumonia and will be assessed at the cluster level.

**Discussion:**

NIGRAAN takes a novel approach to implementation research and explores whether training of LHSs in supervisory skills results in improving the CCM practices of childhood diarrhoea and pneumonia. No significant harm to participants is anticipated. The enablers and barriers towards improved CCM would provide recommendations to policymakers for scale up of this intervention nationally and regionally.

**Trial registration:**

NIGRAAN is registered with the ‘Australian New Zealand Clinical Trials Registry’. Registration Number: ACTRN12613001261707

## Background

Pneumonia and diarrhoea are the leading causes of morbidity and mortality in children under 5, globally [1,2]. Together, these two early childhood illnesses account for one-fifth of the total number of deaths around the globe [3], the major proportion of which occur in the resource poor settings, most notably in Sub-Saharan Africa and South Asia [4-6].

By 2015, the United Nations Millennium Development Goal (UN MDG) 4 aims to reduce under 5 mortality by two-thirds. Pakistan is also a signatory to the UN MDGs and is among those nations who are far behind in achieving this target [7,8]. In Pakistan, around quarter of the children under 5 suffer from diarrhoea each year and one in six children experiences at least one episode of pneumonia annually [9]. These figures translate into around 0.7 million episodes of diarrhoea and more than 0.35 million episodes of pneumonia each year. Ironically, the coverage of proven lifesaving interventions like oral rehydration salt (ORS) for diarrhoea and antibiotics for pneumonia remains quite low [10]. In Pakistan, only 38% of the children receive ORS for diarrhoea and approximately 41% receive antibiotics for suspected pneumonia [9].

A ‘National Programme for Family Planning and Primary Healthcare’ commonly referred to as ‘Lady Health Workers Programme (LHW-P)’, was instituted in 1993–1994 in Pakistan. The programme aimed to provide maternal and child health services to the 60% underserved rural and remote population [11,12]. Under this programme, the women from the local community that met the specified eligibility criteria in terms of education and residence were recruited and trained as ‘lady health workers’ (LHWs) to serve as a ‘focal point of care’ for their respective communities [13]. On an average, an LHW usually serves around 100–150 households [14] translating into a population of approximately 1,000 individuals in her catchment area. The LHW-P also inducted ‘lady health supervisors’ (LHSs) to regularly monitor and supervise the LHWs. Each LHS usually supervises 20–25 LHWs. The LHS conducts random supervisory visits and collects monthly reports from the LHWs which provide information on type of cases encountered and relevant services provided. However, even after two decades, the overall impact on the health status at the population level has been very minimal especially in reducing early childhood disease burden and mortality attributable to diarrhoea and pneumonia.

**Figure 1:**
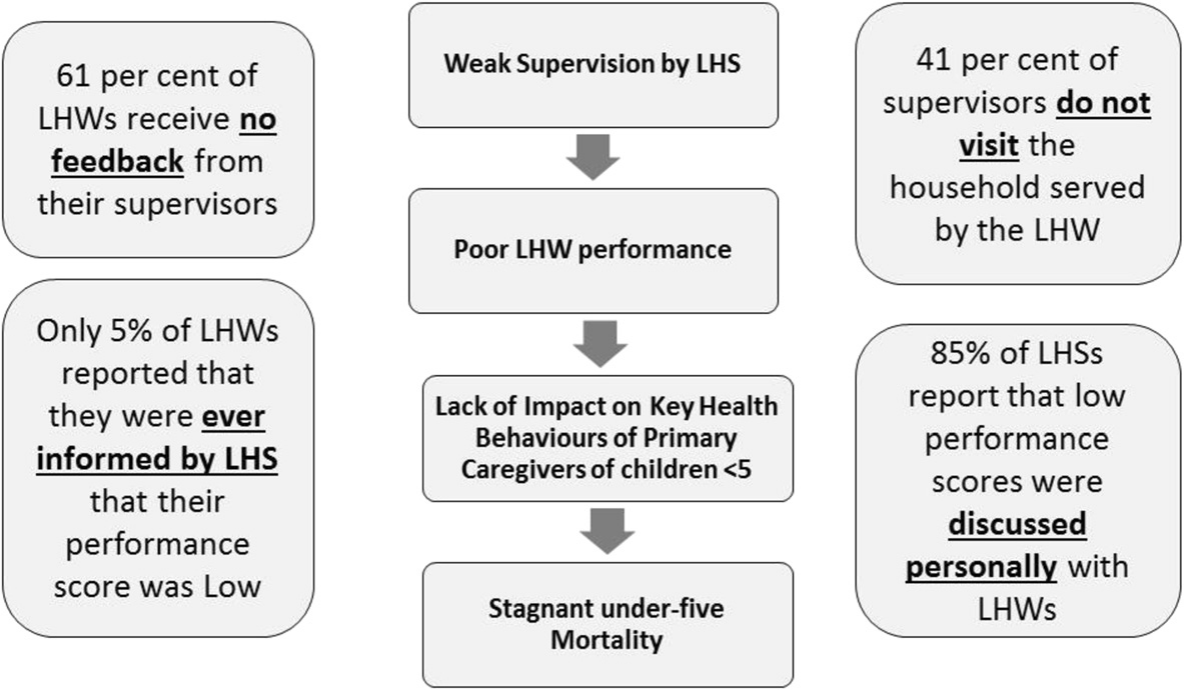
**Issues in LHW programme amenable to intervention [** 15 **].**

The ‘Fourth External Evaluation of National Programme for Family Planning and Primary Health Care’ (FENP) conducted in 2008 provides insight into the causes and determinants of this stagnant mortality rate. Firstly, the LHWs lack skills and are not well prepared in managing cases of pneumonia and diarrhoea in the community. Only 8% LHWs are sensitised to the idea of looking for danger signs in the sick child and merely four out of five can correctly diagnose pneumonia. Secondly, the LHSs too have inadequate supervisory and clinical mentorship skills. Only 40% of LHWs receive the required feedback by their LHS and 59% of LHS pay a supervisory visit to their LHWs catchment area [15]. Figure 1 highlights the issues in the LHW programme which are amenable to the NIGRAAN intervention.

This implementation research project titled ‘NIGRAAN’ (Urdu word, meaning supervision) is designed to bridge these structural gaps in the existing LHW-P. The aim of NIGRAAN is to improve community case management (CCM) of childhood diarrhoea and pneumonia by health workers (LHWs and LHSs) and community caregivers (e.g. mothers) through strengthened supervision and mentorship by LHSs. The project utilises a cluster randomised design with each LHS as one cluster to estimate cluster level changes in CCM due to the intervention. Ultimately, this has the potential to reduce childhood morbidity and mortality due to diarrhoea and pneumonia.

### Primary outcome

The primary outcome is improvement in CCM practices of childhood diarrhoea and pneumonia.

This outcome pertains to the cluster level as the intervention targets LHSs and through them their respective LHWs. It will be achieved through the following effects of the NIGRAAN intervention:

1. Improved knowledge, skills and supervisory processes among LHSs for CCM of pneumonia and diarrhoea in children under 5
2. Improvement in LHW knowledge, skills and performance as a result of structured supportive supervision by LHSs
3. Improved knowledge of community caregivers through interactions with LHWs and LHSs during community management of children with diarrhoea and pneumonia

## Methods

### Study design

**Figure 2:**
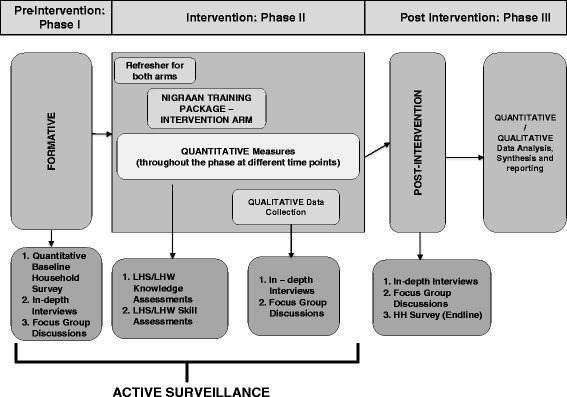
**Overview of NIGRAAN study phases.**

This is a cluster randomised intervention trial consisting of three sequential phases:

1. Pre-intervention: phase I
  Focus group discussions (FGDs) and in-depth interviews (IDIs) explore stakeholders’ (LHS/LHWs/policymaker) perspectives about the structural gaps in the LHW-P and health workers’ basic knowledge and skills related to CCM of childhood diarrhoea and pneumonia, etc. The findings would help to build an optimised intervention addressing deficient areas of knowledge and skills in the next phase. The baseline household survey (administered through a structured questionnaire) has been simultaneously administered to identify community caregivers’ practices related to childhood diarrhoea and pneumonia.
  An active case surveillance with a Global Positioning System (GPS) will also be initiated to identify, track and follow up all reported cases of childhood pneumonia and diarrhoea from the catchment households of randomly selected five LHWs/LHSs in both arms of the study (total 170 LHWs/34 LHS). Upon case identification, this surveillance will help to sample LHS and LHWs for assessment of their knowledge and skills related to CCM of diarrhoea and pneumonia. Knowledge assessment questionnaires (KAQs) and scorecards will be used for this purpose.
  Data obtained through baseline survey, scorecards, KAQs, FGDs and IDIs will be analysed to get consolidated results for the pre-intervention phase.
2. Intervention: phase II
  Phase II incorporates the refresher training of all LHS and execution of NIGRAAN specific intervention package (described below) for 9–12 months. The active case detection continues during this phase for periodic skill assessment of health workers through scorecards and KAQs and to distribute written feedback cards to LHWs as part of the NIGRAAN intervention package.
  Sample FGDs and IDIs will track the uptake of intervention by all stakeholders.
3. Post-intervention: phase III
  Phase III includes an end-line household survey and qualitative data collection through FGDs and IDIs to identify barriers and enablers for NIGRAAN implementation and scale up.
  All the data obtained through the surveys, scorecards, KAQs, FGDs and IDIs during intervention and post-intervention phases will be analysed and results synthesised through method triangulation. Figure 2 provides an overview of NIGRAAN study phases.

### Study setting

District Badin in rural part of Sindh province, Pakistan, was chosen as the study site. It is approximately 4 hours one way by a feasible road drive from Karachi. The district has an operational LHW-P and the Department of Health, Government of Sind agreed to implement the project with the researchers. There are no previous similar projects currently being executed through the LHW-P in Badin. There are 1100 LHWs present in Badin supervised by 36 LHSs.

### Study participants

#### Policymakers and implementers

For the purpose of qualitative assessments at the provincial level, key decision-makers at the Provincial Department of Health and the Provincial Programme Implementation Unit of LHW-P will be approached. At the district level, ‘programme coordinators’ at the ‘District Programme Implementation Unit’ will be the point of focus.

#### Lady health supervisors (LHSs)

An LHS is the immediate female supervisor of LHWs, attached with the first-level care facility (FLCF). She has at least 8 years of schooling and is a local resident with a previous background of having worked as a lady health visitor (LHV) or an LHW. LHSs are recruited to provide supervisory support and ensure quality performance by the LHWs. The LHS collects progress reports, guides and addresses problems raised by LHWs. At the end of each month, the supervisor compiles evaluation reports and submits these to the districts. LHSs fulfilling the following criteria will be involved in project NIGRAAN:

1. LHS who is performing duties as part of LHW programme within geographical boundaries of District Badin
2. LHS whose employment terms are permanent
3. LHS who conducts ‘field monitoring visits’ and reports them to the district level authorities.

LHSs in this study will be involved primarily for periodic assessment of their skills pre and post intervention and in implementing the NIGRAAN intervention by providing written feedback cards to their assigned LHWs.

#### Lady health workers

LHWs are salaried staff, preferably married and educated (minimum 8 years of schooling). They are mostly permanent residents of the area, well known and are familiar with the houses assigned to them in their respective catchment areas. They are trained and supervised by LHSs according to a predefined checklist. A trained LHW provides basic health care to her own community. Each LHW maintains her register, reports to her assigned LHS and submits a monthly report based on specified LHW-P management information tools. The inclusion criteria for LHWs in project NIGRAAN are as follows:

1. LHW who is performing duties as part of LHW programme within geographical boundaries of District Badin
2. LHW who provides services to the households as per LHW programme guidelines and reports the relevant data to the respective supervisor/LHS

LHWs in this study will be involved primarily for periodic assessment of their skills pre and post intervention and in implementing the NIGRAAN intervention by actively reporting cases of pneumonia and diarrhoea to their respective LHSs.

#### Community caregivers

Community caregivers include mothers of children under 5 years of age and/or other caregivers for children under 5, in the population of the study site. The inclusion criterion for community caregivers is:

1. Community caregiver/parent/guardian permanently residing in the household falling under the geographical scope/coverage area of the LHW enrolled into the study
2. Community caregiver residing in a household that has at least one child under 5 years of age

Pre- and post-intervention household surveys will assess their knowledge and practices for management of childhood diarrhoea and pneumonia.

### Sampling

Our sample size is estimated based on the expected number of children under 5 who experience an episode of pneumonia per year. The estimated incidence of pneumonia among children under 5 in Pakistan is 0.41 episodes per child-year (e/cy) [9].

#### Sample estimates

It was estimated that to achieve 80% power for estimating the effect of the intervention at 95% confidence interval and intra-cluster correlation coefficient of 0.122, 17 LHSs (1 LHS = 1 cluster) will be required for intervention and non-intervention arms each.

Five (5) LHWs will be selected in each cluster (85 each in intervention and control arm). These 170 LHWs are required to be able to detect the required number of diarrhoea and pneumonia cases in their catchment households for continuous skill assessment of LHWs and LHSs and to execute the intervention.

The sample for qualitative FGDs was drawn from the above pool of 170 LHWs at random with a target of 8–10 participants in each FGD.

**Figure 3:**
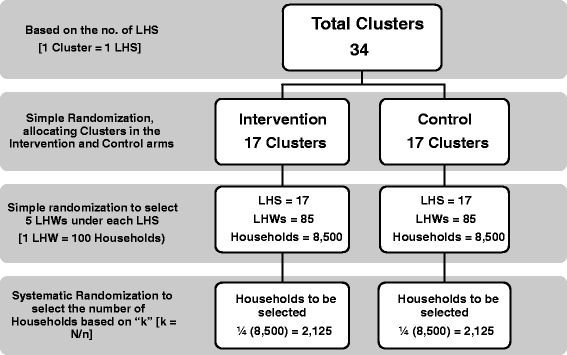
**Overview of NIGRAAN sampling strategy for baseline survey.**

To attain 80% power with 95% confidence interval for the baseline household survey, we estimated that at least 8,500 households will have to be selected in each arm (one LHW covers approximately 100 households). With a systematic randomisation of taking every fourth (4th) household with an expected number of one child under 5 years per household, at least 2,125 households will have to be approached in the baseline/endline survey in each arm to assess community caregiver practices for management of childhood diarrhoea and pneumonia. NIGRAAN baseline survey sampling strategy is depicted in Figure 3.

#### Allocation to intervention

LHSs were allocated to clusters based on a parallel design with 1:1 ratio. The allocation of 34 LHSs to intervention/control arm clusters was blinded at the cluster level and carried out through computer-generated randomised tables in computer software MS Excel. The process was carried out by a person not otherwise involved in the study.

#### Consent

For the baseline survey, written informed consent was sought from the primary caregivers before the initiation of interview.

Informed consent will be sought from all the LHSs and LHWs when the intervention is rolled out.

#### Blinding

After assignment to intervention and control clusters, all participants, field staff, independent evaluators (IEs), and data entry personnel will be blinded. For this purpose, control and intervention cluster LHSs and LHWs will be assigned study IDs with the key available only to the core Aga Khan University research team.

### The intervention: NIGRAAN training package

The NIGRAAN intervention applies at the cluster level. Each of the LHSs, the primary targets of the intervention, constitutes a cluster. Through the LHSs, the intervention targets LHWs at the cluster level too.

The intervention consists of the following components:

#### A. Training

In a 4-day training workshop, a training consultant would facilitate the 17 LHSs in the intervention arm to improve their theoretical and practical understanding of management of childhood diarrhoea and pneumonia and clinical mentoring and supervision skills. Special focus would be to improve LHS skills in providing verbal and written feedback to their assigned LHWs. The face-to-face learning would be complemented by hands-on training in the hospital setting along with practical exercises in a simulated environment. The instructional methodologies used during the training would include interactive lectures complemented with audio-visual aids/videos (to be sourced through WHO) and role plays, etc. Prior to the NIGRAAN specific training (described above), a 2-day refresher training based on all aspects of existing LHW curriculum would be offered by master trainers (trained through the project) for all the 34 LHS in both arms.

#### B. Supervisory tools

To complement the existing LHW Management Information System, NIGRAAN intervention will also introduce the following tools:

1. The ‘Modified Supervisory Checklist’ has been adapted from the existing LHW-P Management Information System kit for LHSs. It augments certain components to strengthen the monitoring of community case management of LHWs by LHSs and adds the feature of direct observation.
2. The ‘Supervisor’s Tally Sheet for Compilation of Quality of Case (QoC) Management’ is the consolidated version of the modified supervisory checklist providing at a glance view of the progress and quality of case management as well as picks up the gaps in case management practices of LHWs working under a given LHS.
3. The ‘LHS Feedback Card’ for individual LHWs is the most important tool in the intervention package. It aims to introduce the practice of written feedback to be provided to LHWs through LHSs.

**Figure 4:**
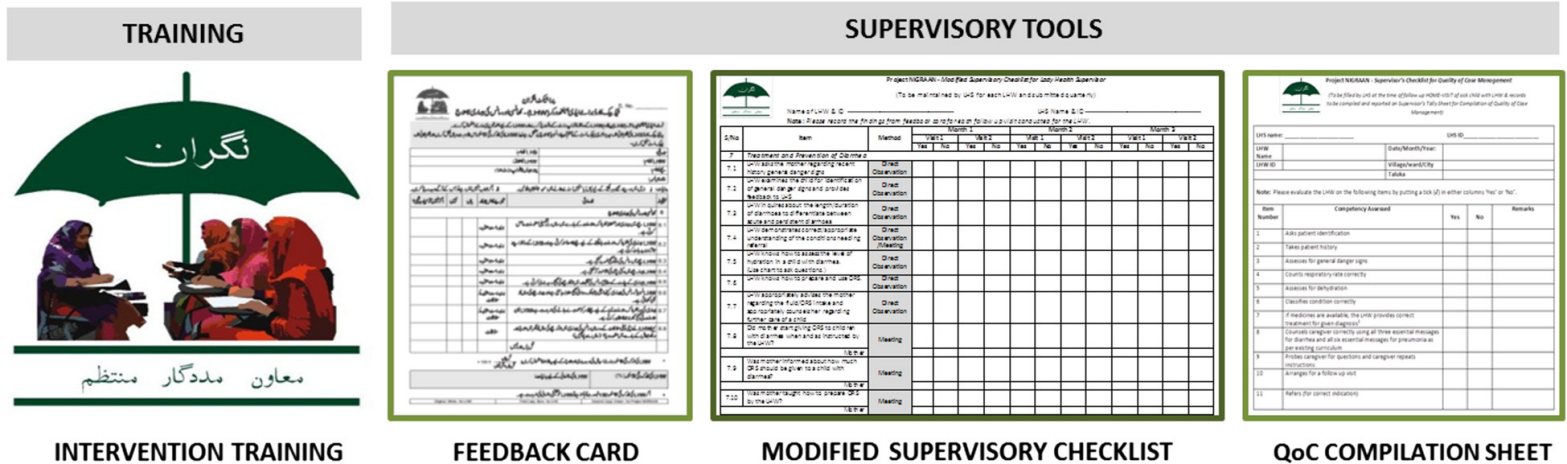
**NIGRAAN intervention package.**

Various components of NIGRAAN intervention package are shown in Figure 4.

#### Potential harms and mitigation

We do not anticipate any risk to primary caregivers of children under 5. However, whilst NIGRAAN is feeding seamlessly into an already existing government system and is not deviating from existing LHW/LHS charter of duties, it is remotely possible that LHWs and LHS may experience higher levels of work-related stress and pressure. FGDs will be conducted regularly to assess the feasibility of continuing the intervention. If self-reported work pressure is noted and some LHSs feel overburdened and stressed out as a result of ‘a new way of doing the same things’ i.e. written instead of a verbal feedback and more stringent clinical mentoring of LHWs by LHS for the management of pneumonia and diarrhoea, stress-mitigating steps such as counselling and supportive consultation efforts will be taken in those clusters where such a stress is experienced. On-ground counselling will also be offered by the research team, to LHWs reporting increasing stress and difficulty in coping.

### Data collection instruments

Quantitative instruments have been developed to collect the appropriate population level data from the community as well as to measure the performance of the health workers—LHSs and LHWs—on the knowledge and skills domains. On the other hand, qualitative instruments will explore the structural gaps in the LHW-P and health workers’ basic knowledge and skills related to CCM of childhood diarrhoea and pneumonia.

#### Quantitative instruments

1. Skills assessment scorecard A: lists the variables for assessing clinical case management skills of LHWs and LHSs. Each item on the scorecard is linked with the essential tasks related to CCM of pneumonia and diarrhoea in children under 5.
2. Skills assessment scorecard B: will be used for assessing supervisory and clinical mentorship skills of an LHS. This will incorporate elements of clinical mentoring and supportive feedback.
  ‘Independent evaluators’ with relevant background and training in community health and clinical care will be recruited to accompany the health workers during her home visits and fill the scorecards.
3. Household survey questionnaire: will be used for baseline and end-line household survey to collect data on caregiver knowledge and practices.
4. Knowledge assessment questionnaire: will be used to assess LHS and LHW knowledge regarding CCM of pneumonia and diarrhoea in children under five. This instrument, in addition to being deployed pre intervention, will also be used periodically to assess change in LHS and LHW knowledge and skills.
  Table 1 provides an overview of the various quantitative tools.

**Table 1:** **Overview of NIGRAAN quantitative tools**

**Tools/instruments**	**Purpose**	**User(s) in the field**
*Household survey questionnaire*	To record the socio-demographic information and caregiver practices regarding diarrhoea and pneumonia of the population under study as well as to document the morbidity due to diarrhoea and pneumonia	*The data collection team*
*Knowledge assessment questionnaire*	To assess the theoretical understanding and knowledge of LHSs and LHWs regarding community case management of diarrhoea and pneumonia	*The research team*
*Skills assessment scorecard ‘A’*	To assess the practical/clinical skills of LHSs and LHWs regarding community case management of diarrhoea and pneumonia	*Independent evaluator(s)*
*Skills assessment scorecard ‘B’*	To assess the supervisory and clinical mentoring skills of LHSs in terms of providing feedback and supportive supervision to LHWs	*Independent evaluator(s)*

#### Qualitative instruments

1. In-depth interview questionnaire:
  This semi-structured questionnaire will be used to guide the interviews with key informants in all phases of project NIGRAAN. This questionnaire uses open-ended questions to explore stakeholder knowledge about childhood pneumonia and diarrhoea, perspectives and experiences of health workers regarding supportive supervision and their experience and perceptions about the utility of NIGRAAN intervention.
2. Focus group discussion guide:
  This tool will be used by FGD moderators to guide discussions. It consists of key probes and triggers to guide the discussion.

Table 2 provides an overview of major NIGRAAN assessments and activities, the relevant study instruments, and the timelines. The table also correlates the intended outcomes of the study with these assessments and time points of measurement.

**Table 2:** **Relevance of NIGRAAN outcomes with the major assessments, relevant study instruments and timelines**

**Outcomes**	**Measurements**	**Timepoints**
*Primary outcome:*	Baseline, and end-line household surveys to assess change in community caregiver practices for management of childhood diarrhoea and pneumonia	*Baseline:* at 4–6 months
*Improvement in community case management (CCM) practices of diarrhoea and pneumonia*		*End-line:* at 20–22 months
*Secondary outcome 1:*	*Qualitative tools:*	*Qualitative tools*
*Improved perceptions, knowledge and skills among LHSs for community case management of pneumonia and diarrhoea in children under 5*	Focus group discussions (FGDs) and in-depth/key informant interviews	Once in each of the three phases of the project
	*Quantitative assessments:*	*Quantitative assessments*
	Knowledge assessment questionnaire and skills assessment scorecards ‘A’ and ‘B’	KAQs and scorecards: phases I and II on a periodic basis.
*Secondary outcome 2:*	*Qualitative tools*	*Qualitative tools*
*Improvement in LHWs perceptions, knowledge, skills and performance as a result of ‘structured supportive supervision’ by LHSs*	Focus group discussions (FGDs) and in-depth/key informant interviews	Once in each of the three phases of the project
	*Quantitative assessments:*	*Quantitative assessments*
	Knowledge assessment questionnaire and skills assessment Scorecards ‘A’ and ‘B’	KAQs and scorecards: phases I and II on a periodic basis.

### Plan of data analysis

All quantitative data would be primarily analysed in IBM SPSS version 19. Changes in community and health worker knowledge and case management practices of diarrhoea and pneumonia pre and post intervention will be compared between the intervention and control arms. Tests of significance will be performed.

## Trial status

Pre-intervention phase has concluded. Preparation for installing surveillance is on-going so that cases can be identified for starting the intervention. Recruitment in control and intervention arm of the study will start at the end of November 2014 and continue for the next 9–12 months.

## Discussion/conclusion

NIGRAAN is an implementation research project using a management intervention to improve CCM of childhood pneumonia and diarrhoea. It is different from other randomised trials using top-down approaches to deliver interventions as it works within the current structure of LHW-P in Pakistan. NIGRAAN has actually tried to bridge the know-do gap in the current LHW-P of Pakistan by trying to address one of the structural gaps i.e. lack of supportive supervision by LHSs [15].

There is evidence from other studies that increased supervisory competence and supportive supervision improves knowledge, skills and overall performance of junior staff [16,17]. Adequate supportive supervision of community health workers has also been reported to be a crucial factor for ensuring effectiveness of weak health systems [18]. Moreover, evidence from low- and middle-income countries suggests that for CCM to be successful, health workers need to be adequately supervised [19].

Joint statements from WHO and UNICEF also recommend that countries should support strategies aimed at adequately supervising community health workers’ pneumonia case management activities and develop suitable strategies to educate health workers at all levels about case management of diarrhoea using ORS and zinc supplements [20,21].

Regarding study limitations, there is a possibility that being observed during field duties may cause a slight variation in LHW performance leading to an underestimation or overestimation of performance.

The LHW-P in Badin is quite representative of the programme at the provincial and national level in terms of infrastructure and functionality. Therefore, results could be useful for programme implementers and workers across the country.

Findings from NIGRAAN would therefore be of significance in exploring whether training of LHSs in supervisory skills results in improving the CCM practices of childhood diarrhoea and pneumonia. The enablers and barriers towards the uptake of this supervisory intervention would provide recommendations to policymakers for further scale up nationally and regionally.

NIGRAAN is funded by the World Health Organization, Geneva, Department of Maternal, Newborn, Child and Adolescent Health. The trial is registered with the ‘Australian New Zealand Clinical Trials Registry’. Registration Number: ACTRN12613001261707

## Abbreviations

MDG: Millennium Development Goal
LHW: lady health worker
LHS: lady health supervisor
FENP: Fourth External Evaluation of National Programme
CCM: community case management
FGD: focus group discussion
IDI: in-depth interview
FLCF: first-level care facility
LHV: lady health visitor
WHO: World Health Organization
QoC: quality of case
